# Morphological brain differences between adult stutterers and non-stutterers

**DOI:** 10.1186/1471-2377-4-23

**Published:** 2004-12-10

**Authors:** Lutz Jäncke, Jürgen Hänggi, Helmuth Steinmetz

**Affiliations:** 1 Institute of Psychology, Department Neuropsychology, University Zurich, Switzerland; 2 Department of Neurology, Johann-Wolfgang Goethe University Frankfurt am Main, Germany

## Abstract

**Background:**

The neurophysiological and neuroanatomical foundations of persistent developmental stuttering (PDS) are still a matter of dispute. A main argument is that stutterers show atypical anatomical asymmetries of speech-relevant brain areas, which possibly affect speech fluency. The major aim of this study was to determine whether adults with PDS have anomalous anatomy in cortical speech-language areas.

**Methods:**

Adults with PDS (n = 10) and controls (n = 10) matched for age, sex, hand preference, and education were studied using high-resolution MRI scans. Using a new variant of the voxel-based morphometry technique (augmented VBM) the brains of stutterers and non-stutterers were compared with respect to white matter (WM) and grey matter (GM) differences.

**Results:**

We found increased WM volumes in a right-hemispheric network comprising the superior temporal gyrus (including the planum temporale), the inferior frontal gyrus (including the pars triangularis), the precentral gyrus in the vicinity of the face and mouth representation, and the anterior middle frontal gyrus. In addition, we detected a leftward WM asymmetry in the auditory cortex in non-stutterers, while stutterers showed symmetric WM volumes.

**Conclusions:**

These results provide strong evidence that adults with PDS have anomalous anatomy not only in perisylvian speech and language areas but also in prefrontal and sensorimotor areas. Whether this atypical asymmetry of WM is the cause or the consequence of stuttering is still an unanswered question.

## Background

Persistent developmental stuttering (PDS) is a relatively severe disturbance characterized by involuntary, audible or silent, repetitions or prolongations of sounds or syllables. These are not readily controllable and often are accompanied by other movements and by negative emotions [1,2]. Developmental stuttering evolves before puberty without apparent brain damage or other known cause. Several authors suppose a hereditary component of PDS because of the relatively high concordance rate in family members of PDS subjects (70% for monozygotic twins, about 30% for dizygotic twins, and 18% for siblings of the same sex) [3-5]. Because of this hereditary component and the early onset of stuttering it has repeatedly been suggested that some kind of anatomical or neurophysiological predetermination increases the vulnerability for stuttering [for a summary of theories and findings related to stuttering research [6]].

Several experimental studies have shown that stutterers reveal prolonged manual and vocal reaction times to simple and complex verbal and nonverbal stimuli [7-10], reduced bimanual coordination measures [11-13], atypical functional lateralizations [14-18], abnormalities in the auditory system [19-21], or increased variability of time-critical speech parameters [9,22,23]. More recent neuroimaging studies have shown atypical hemodynamic responses in speech-related brain areas even during fluent utterances suggesting a dysfunctionally operating speech control circuit in stutterers [24-29]. However, although much research has been invested to understand the neurophysiological mechanisms and underpinnings of this disorder, none of the aforementioned studies provide a substantial breakthrough in understanding stuttering. Several researchers have hypothesized subtle but nevertheless crucial deficiencies in the anatomical and neurophysiological underpinnings of the speech and language system. One popular hypothesis is that stutterers would show an atypical lateralisation of the speech system (reversed or reduced laterality) thought to make the system more vulnerable to speech dysfluencies [17]. Although recent neuroimaging studies have shown atypical activation and deactivation of brain regions in adults with PDS [24,30-32] the anatomical underpinnings of stuttering have not been examined in detail so far.

According to the present literature four anatomical studies revealed brain abnormalities in stutterers compared to controls. The earliest study examined two left-handed stuttering siblings using CT and revealed an atypical (reduced) anatomical asymmetry of the occipital poles [33]. The first high-resolution MRI study investigating stutterers revealed a reduced volumetric asymmetry of the planum temporale (a brain area which is involved in higher order auditory processing) and other anatomical peculiarities in speech-related areas [34]. A more recent paper of the same group revealed that PDS is also associated with atypical (mostly reduced) prefrontal and occipital lobe asymmetries [35]. In addition, deficits in language processing were associated with some anatomic measures in the adults who stutter. Using a new MRI technique (diffusion tensor imaging: DTI), that allows the assessment of white matter ultrastructure, Sommer et al. [36] found an area of decreased white matter tract coherence in the left Rolandic operculum. This structure is adjacent to the primary motor representation of tongue, larynx, and pharynx and the inferior arcuate fascicle linking temporal and frontal language areas, which both form a temporo-frontal language system involved in word perception and production. Thus, there are indeed first strong hints that the brain of stutterers differ from non-stuttering subjects on a macroanatomical level suggesting that morphological predispositions determine stuttering.

Although the aforementioned anatomical studies have focussed on the anatomical foundations of stuttering, several questions are not answered yet. Therefore we re-examined the hypothesis of anatomical differences between stutterers and non-stutterers using voxel-based morphometry (VBM). This approach circumvents the problem of analyzing predetermined regions of interests by analyzing stereotactically normalized brains on a voxel-by-voxels basis with respect to differences in the volume of white matter (WM) or grey matter (GM) [37,38]. This approach has successfully been used in the last 8 years for several clinical populations and allows studying the morphology separately for GM and WM looking at the entire brain [39-41]. A further advantage of this method is the objectivity and thus rater independence. We hypothesized that beside the previously reported atypical anatomical asymmetries in perisylvian and frontal areas there should be additional differences in further brain areas also involved in speech motor control. Because several studies report that the auditory system in stutterers is dysfunctional [19,21,42-47] especially during speaking (thus, emphasizing the role of auditory feedback in the context of stuttering), we anticipated structural peculiarities in the auditory cortex (Heschl's gyrus and the planum temporale) in this group. In addition, we also anticipated anatomical peculiarities in frontal brain areas and in the somatosensory and motor system controlling the speech muscles.

## Methods

### Subjects

The sample included adults with PDS (n = 10) and controls (n = 10) matched according to sex, age and education. All subjects were consistent right-handers (CRH) according to the Annett handedness questionnaire (AHQ) [66]. Our sample contained the approximate sex distribution as those reported in population studies of adults who stutter; thus, there were more men (n = 8) than women (n = 2) in this sample. The dysfluent sample was limited to adults with PDS who had been diagnosed with developmental stuttering before the age of 8 years and had undergone treatment at some point, but continued to be dysfluent. None of the subjects was taking centrally acting medications that could have resulted in PDS, and all met the clinical criteria of developmental stuttering – not acquired stuttering. Of the adults who stuttered, 50% had a family history of stuttering; none of the controls had a family history of stuttering. Stuttering severity was determined using the Stuttering Severity Inventory (SSI) [67] with individuals in the sample ranging from mild (2), moderate (7) to severe (1). All participants were native German speakers with no reported history of dyslexia, specific language impairment, attention deficit disorder, traumatic brain injury, substance abuse, or other neuropsychiatric conditions. All participants gave informed consent before participating.

### MRI scanning protocol and data analysis

We used a Siemens 1.5 T magnet and a 22-min fast-low-angle-shot MR sequence yielding 128 contiguous sagittal slices with 1 × 1 × 1.17 mm image voxel size [68–70]. Data were analyzed on a PC workstation using MATLAB 5.3 (MathWorks, Natick, MA) and SPM 99 (Wellcome Dept. Cogn. Neurol, London; ) [71]. Preprocessing were guided by the VBM method proposed by Godd et al. [40,41]. In short, the following steps were conducted: (1) Spatial normalization of each brain to the MNI space using the MNI template; (2) spatial smoothing with an 8-mm full-width at half-maximum (FWHM) isotropic Gaussian kernel; (3) creating of a mean anatomical image from these normalized and smoothed scans; (4) stereotactic normalisation of all MRI scans (in native space) using the newly developed template and non-linear smooth spatial basis functions; (5) these spatially normalized images were resliced with a final voxel size of 2 × 2 × 2 mm^3^. The normalized scans were then segmented into grey (GM) and white matter (WM), cerebro spinal fluid (CSF), and other non-brain partitions applying the algorithmus implemented in SPM99 based on the algorithms developed by Ashburner and Friston [37,38]. In order to sensitize our subsequent statistical analysis not only to differences in the GM (WM) proportions but also to differences in the true GM (WM) volumes a further processing step – known as the 'Jacobian Modulation – was incorporated. The partitioned images (GM and WM) were multiplied by the Jacobian determinants of the deformation field transforming the GM and WM density values into volume equivalents [38,40]. The normalized, segmented (and modulated) images are smoothed using a 10-mm FWHM isotropic Gaussian kernel to improve statistical quality of the data (e.g., normal distribution).

### Statistical analysis of VBM data

The normalized, smoothed, segmented (and modulated) data were analyzed using statistical parametric mapping (SPM99) employing the framework of the General Linear Model. Regionally specific differences in GM (and WM) (both for the density and the volume equivalents) between groups were assessed statistically using a two-tailed contrast. Corrections for the search volume (and implicit multiple comparisons) in terms of the P values were made using Gaussian random field theory, which accommodates spatial correlations inherent in the data and is now established as the conventional approach to inference in smooth spatially extended data. We restricted the search volume to the GM or WM volume enabling us to increase the statistical power of statistical testing. Significance levels for two-sided T statistics were set at T = 5 (corrected for multiple comparisons across the WM or GM volumes) and a spatial extend criterion of k = 50. The spatial extend of k = 50 was introduced because this volume size roughly corresponds to the size of a meaningful anatomical area (0.4 cm^3^).

## Results

Because there was no substantial difference between the results of our statistical tests for the density and volume equivalents, we only report the findings based on the analysis of the volume equivalents. We found increased WM volumes in stutterers within four clusters on the right hemisphere. The clusters are located in the superior temporal gyrus (STG) including the planum temporale, the precentral gyrus (PrCG), the inferior frontal gyrus (IFG) comprising the pars opercularis (POP), and the middle frontal gyrus (MFD) (Table 1). There was no significant difference between stutterers and non-stutterers with respect to the GM volumes.

**Table 1:** Regions of increased WM volumes in stutterers. Indicated are the peak differences (in t-values), their stereotactically coordinates, and the associated anatomical labels derived from the MNI standard brain. Please note, there were no areas with increased WM volumes in controls compared to stutterers.

**Anatomic region**	**Coordinates (X, Y, Z)**			**t-Value**
R Superior temporal gyrus (STG)	64	-34	21	7.45
R Inferior frontal gyrus (IFG)	66	8	21	6.58
R Middle frontal gyrus (MFG)	44	48	11	6.23
R Precentral gyrus (PrCG)	30	-28	63	7.25
R Precentral gyrus (PrCG)	62	-12	37	6.53

**Figure 2:**
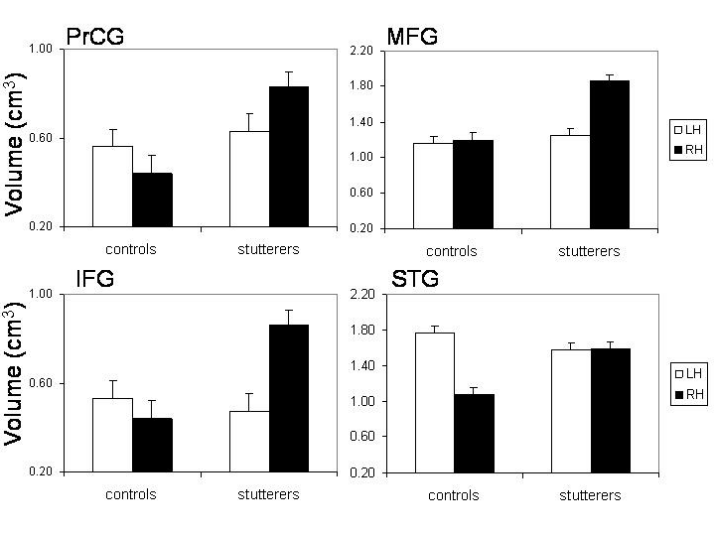
**ROI analysis **Mean WM volumes (and standard errors of the mean as vertical bars) in the precentral gyrus (PrCG), middle frontal gyrus (MFG), inferior frontal gyrus (IFG), and the superior temporal gyrus (STG) broken down for the left (open bars, LH) and right (filled bars, RH) hemisphere. The STG comprises Heschl's gyrus and the planum temporale. The volume measures are expressed as arbitrary values because these measures were obtained from brains transformed into the MNI space.

In order to understand the differences between stutterers and non-stutterers with respect to the WM volumes in these anatomical areas more precisely, we placed regions of interest (ROI) in these anatomical areas and the homotopic areas on the left hemisphere. For the auditory cortex we used a rectangular ROI including Heschl's gyrus (HG) and the planum temporale (PT) (size of the ROI on both hemispheres: 8.4 cm^3^). The placement pf these ROIs were guided by anatomical landmarks and published probability atlases of the HG and PT [48,49]. The other ROIs were defined according to the stereotactic coordinates found in the VBM analysis. For these ROIs, rectangular volumes (10 mm edge length resulting in a volume of 10 × 10 × 10 mm) were used. The mean WM measures were calculated for each ROI and subjected two-way ANOVAs with one repeated measurement factor (Hemisphere: left vs. right) and one grouping factor (Group: stutterers vs. non-stutterers). Because we found significant interaction effects for all ROIs we will only interpret these interactions. For the auditory cortex we found a strong main effect for the factor Hemisphere (F(1, 18) = 29.2, p <= 0.001, ETA^2 ^= 0.62) and a significant interaction between both factors (F(1, 18) = 31.6, p <= 0.001, ETA^2 ^= 0.64). Subsequent Scheffé contrasts and Figure 2 show that there is a strong between-hemisphere difference for non-stutterers (larger WM volume on the left hemisphere, p < 0.01) but not for stutterers (p > 0.4). For the IFG there were strong main effects (Hemisphere: F(1, 18) = 9.2, p = 0.007, ETA^2 ^= 0.34; Group: F(1, 18) = 23.6, p <= 0.001, ETA^2 ^= 0.58) and a significant interaction (F(1, 18) = 23.9, p <= 0.001, ETA^2 ^= 0.57). The strong interaction is qualified by a between-hemisphere difference found for stutterers (with larger WM volumes on the right compared to the left IFG) while there is no between-hemisphere difference in non-stutterers. For the PrCG we found a significant between-group difference (F(1, 18) = 11.1, p = 0.004, ETA^2 ^= 0.38) and a significant interaction (F(1, 18) = 31.0, p <= 0.001, ETA^2 ^= 0.64). The pattern of this interaction resembles the interaction found for the IFG with larger WM volumes on the right hemisphere for stutterers than on the left while non-stutterers show similar values for both hemispheres. For the MFG all two main effects as well as the interaction were strongly significant (Hemisphere: F(1, 18) = 23.5, p <= 0.001, ETA^2 ^= 0.56; Group: (F(1, 18) = 13.2, p = 0.002, ETA^2 ^= 0.42; interaction: (F(1, 18) = 18.4, p <= 0.001, ETA^2 ^= 0.50). The interaction is due to the fact that stutterers revealed larger WM volumes on the right compared to the left hemisphere.

In addition, we did not find any correlation between the stuttering severity measures (SSI measures) and the anatomical peculiarities neither in the context of the VBM nor the ROI analysis.

## Discussion

This study was motivated by the question whether stutterers reveal morphological brain anomalies compared to non-stuttering controls. In fact, we found prominent increases of WM in stutterers within a right-hemispheric network including brain structures relevant for language and speech. These areas comprise the STG (including the auditory areas PT and HG), the IFG (including the pars opercularis which is part of Broca's right-sided homologue), the somatosensory area (including the face and mouth representation, as well as the mesial part of the hand representation), and the middle frontal gyrus (MFG). Our findings of regionally increased right-hemispheric WM in stutterers might suggest an increased and possibly atypical intrahemispheric communication within these areas via association fibres [50,51] possibly accompanying different processing strategies in the right hemisphere in stutterers.

Three of the brain areas with different WM composition in stutterers are known to be involved in different speech and language functions. For example, the right IFG (including the pars opercularis) is involved in the perception and generation of phonological or prosodic speech features [52-55] while the ventral part of the precentral gyrus is part of the somatosensory representation of the mouth and tongue. The MFG has been shown to be involved during rhyme and tone perception [34]. The core region of this network is the auditory cortex with neurons specialized for tone, pitch, and prosody perception [56,57]. Within this circuit the auditory cortex plays a pivotal role because speech production follows the ultimate goal to generate speech sounds others can understand. The auditory cues in speech production are either phonetic cues (such as voice onset times or formant transitions) or specific suprasegmental features like duration, intensity, linguistic or emotional stress. During speech production the auditory system controls whether the appropriate auditory cues have been generated by means of auditory feedback control of the own speech. Several studies have shown that the auditory cortex is strongly involved in the continuous control of self-generated suprasegmental speech features (duration, intensity, stress pattern) and that this auditory feedback control is detrimental in stutterers [19-21,45,46,58-60].

The auditory cortices in the two hemispheres are relatively specialized in normal subjects [56,57]. Thus, temporal resolution is better in left auditory cortical areas and spectral resolution as well as processing of prolonged auditory information is better in right auditory cortical areas. It is thought that this functional specialisation is based on cytoarchitectonic peculiarities (more heavily myelinated axons and greater interconnectivity) and the relative composition of WM and GM in this area. In fact, in addition to the present findings, two previous studies [61,62] found a leftward asymmetry of WM volume in the auditory cortex in healthy subjects. However, our findings show that stutterers do not reveal the typical leftward asymmetry; they rather show symmetry with an atypically enlarged WM volume in the right auditory cortex. This atypical symmetry of WM volume in the auditory cortex in stutterers might suggest different and perhaps deficient processing of slowly changing auditory cues necessary to control suprasegmental features. In fact several studies have shown that stutterers reveal substantial peculiarities with respect to various aspects of the auditory feedback of their own speech especially when they control suprasegmental speech features [19,60,63].

The reported morphological features complement previous morphological studies comparing stutterers and non-stutterers. Firstly, this study shows again that stutterers reveal atypical anatomical lateralisation in speech-relevant areas. In three areas (PrCG, MFG, and IFG) stutterers reveal more WM volumes on the right than on the left. For the auditory cortex (STG) we found symmetric WM volumes while non-stutterers typically show a leftward asymmetry for this measure. Thus, some kind of hemispheric imbalance seems to be related to persistent developing stuttering. Secondly, using a different method than Foundas et al. [34], we also found an atypical anatomical lateralisation in the auditory cortex expressed as an increased symmetry of WM volume. Thus, the different "hardware" composition of the auditory cortex in stutterers is a crucial peculiarity possibly determining the processing mode of the right auditory cortex and the interaction between both auditory cortices. Taken together, the present results and findings of previous behavioural and neuroimaging studies emphasize a specific role of the auditory cortex in stuttering. Thirdly, while Foundas et al. [35] found atypical anatomical lateralisation in prefrontal areas we detected increased WM volumes in the right anterior MFG which is part of the prefrontal cortex. Thus, the atypical prefrontal lateralization may be due to atypical lateralisation of the WM volume of the right MFG. Finally, our analysis also revealed an atypical asymmetry with respect to the WM volume in vicinity of the right sensorimotor cortex (including the face and mouth representation as well as parts of the hand representation) possibly suggesting that these areas use different processing strategies as compared to non-stuttering subjects. However, although we and others found large morphological differences between stutterers and non-stuttereres we cannot rule out the possibility that the anatomical differences are the consequence of stuttering rather than the cause. Persistent developmental stuttering commences early in life forcing the affected subject to cope with this annoying and detrimental situation. Thus, some kind of adaptation or cortical reorganisation might accompany this process. Indeed, several studies indicate that intensive practise of various skills might affect the brain even on the macroanatomical level [64,65]. Future studies, however, are clearly needed to disentangle whether the anatomical peculiarities in stutterers are the cause or the consequence of stuttering.

## Conclusions

These results provide strong evidence that adults with PDS have anomalous anatomy not only in perisylvian speech and language areas but also in prefrontal and sensorimotor areas. These anatomical features might indicate a deficiently working speech system. Whether this atypical asymmetry of WM is the cause or the consequence of stuttering is still an unanswered question.

## Competing interests

The author(s) declare that they have no competing interests.

## Authors' contributions

L.J., J.H. and H.S. conceived the experiment and drafted the manuscript. L.J. and J.H. prepared the exact experimental setup. H.S. supervised data acquisition. J.H. and L.J. performed all data and statistical analyses. All authors read and approved the final manuscript.

**Figure 1:**
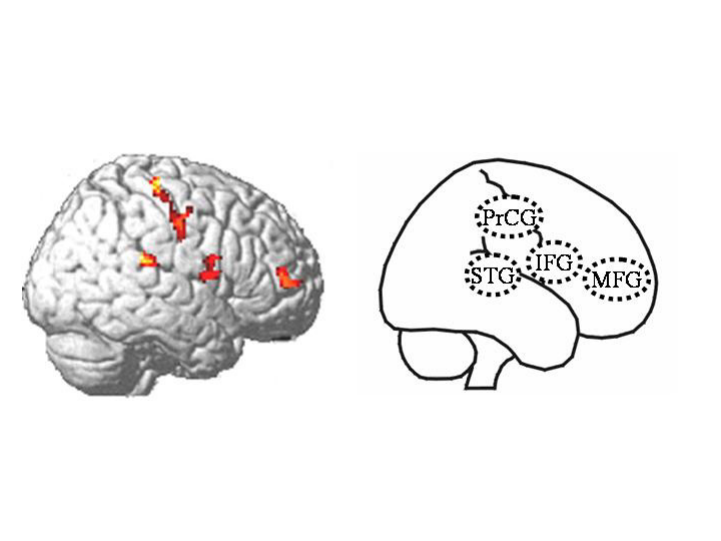
**VBM results **Areas where stutterers show increased relative white matter (WM) volume superimposed onto the standard MNI template. The brain outline on the right indicates the four different anatomical regions showing increased WM volume in stutterers.

## Pre-publication history

The pre-publication history for this paper can be accessed here:


